# Locking Plate Alone versus in Combination with Two Crossed Kirschner Wires for Fifth Metacarpal Neck Fracture

**DOI:** 10.1038/srep46109

**Published:** 2017-04-05

**Authors:** Hongyi Zhu, Zhengyu Xu, Haifeng Wei, Xianyou Zheng

**Affiliations:** 1 Department of Orthopaedic Surgery, Shanghai Jiaotong University Affiliated Sixth People’s Hospital, Shanghai, 200233, China

## Abstract

Fracture of fifth metacarpal neck commonly requires open reduction and internal fixation. However, the current methods of internal fixation in fifth metacarpal neck fractures remain unsatisfactory. Patients with fractures of fifth metacarpal neck received open reduction and internal fixation with either locking plate in combination with two crossed Kirschner wires (K-wires) or locking plate alone were evaluated for the clinical outcomes. Clinical outcomes included grip strength, Michigan hand outcomes questionnaire (MHQ), final angulation and range of motion (ROM) one year after treatment. The averages of MHQ scores, final angulation and ROM of fifth metacarpophalangeal joint of plate with K-wire group were more superior to those of plate group (MHQ 96.7 versus 86.6, final angulation 11.8 versus 23.6, ROM 83.3 versus 72.2). The grip strength had no significant difference between two groups. Locking plate in combination with two crossed K-wires is a more optimal method of fixation compared with locking plate alone.

The fracture of fifth metacarpal neck is a common type of hand injury associated with high-energy trauma. Fifth metacarpal neck fracture accounts for approximately 20% of all hand fractures^1^. In the past, the fractures of fifth metacarpal fractures were commonly treated conservatively. In recent years, published reports were prone to reduction with operative stabilization. A biomechanical study showed that 30 degrees were the upper limit for acceptable final angulation^2^. Therefore, some surgeons use a fracture angle greater than 30° as a relative surgical indication for fifth metacarpal neck fracture^3^,^4^,^5^. A range of surgical techniques have been described for the treatment of fifth metacarpal neck fracture: intramedullary K-wires^5^, transverse K-wires^4^, tension band^6^, locked intramedullary nailing^7^, external fixation^8^, and locking plate fixation^9^,^10^. However, there was no consensus on the most ideal method of fixation. Secondary angulation after surgery was a major complication observed in all current methods of fixation, which limited the efficacy of treatment. We hypothesized that insertion of two crossed K-wires after placement of locking plate could increase the strength of fixation, prevent the secondary displacement, and therefore improve the clinical outcomes. In this study, we compared the clinical outcomes of locking plate with and without crossed K-wire fixation in fifth metacarpal fracture.

## Materials and Methods

The study was approved by the Ethics Committee of Shanghai Jiaotong University Affiliated Sixth People’s Hospital. Informed consent was obtained from all donors. All study methods were in accordance with the Declaration of Helsinki. Acute fifth metacarpal neck fractures (≤3 days) treated with open reduction and lock plate fixation with or without two crossed K-wires were included for analysis from May 2011 to May 2015. The inclusion criteria were completion of at least one-year follow-up, age ranged from 20 to 60. Patients with any of the following criteria were excluded: any injuries on tendons, ligaments, vessels and nerves on the upper limbs, fractures on the upper limbs in addition to fifth metacarpal neck, open fractures.

**Figure 1:**
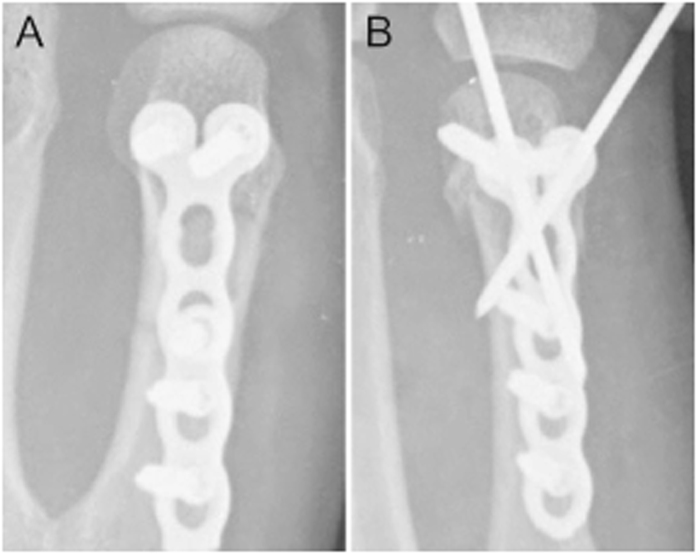
Representative postoperative radiographs of (**A**) locking plate alone and (**B**) locking plate in combination with two K-wires on antero-posterior view.

All patients were operated through a longitudinal dorsal approach facing the fifth metacarpal axis. After reduction and placement of locking plate, two crossed K-wires were inserted or not based on surgeons’ preferences. Two crossed K-wires were inserted dorsally and percutaneously. Surgeries in this study were conducted by 19 specialists of hand surgery. The representative postoperative radiographs were shown in Fig. 1. There was no postoperative immobilization, and active mobilization was encouraged immediately after surgery. Patients were then regularly followed up.

**Figure 2:**
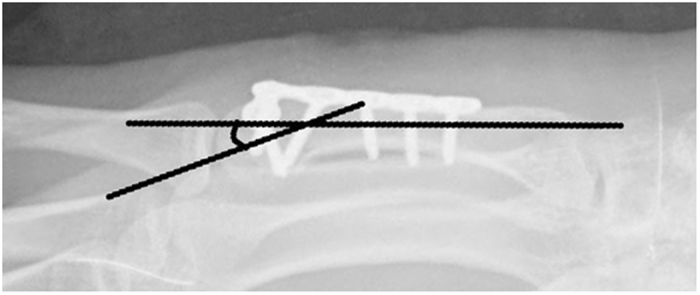
Method of angulation measurement.

Demographic parameters including age and gender were recorded for each group. Assessments were conducted at 1 year after treatment by a trained researcher who was unaware of the details of the patients’ treatment. Clinical assessments included grip strength, Michigan hand outcomes questionnaire (MHQ)^11^. Grip strength was measured with the elbow flexed at 90 degree and the forearm in neutral rotation. Values are expressed as percentages of the values of the contralateral hand. The MHQ is a 37-item questionnaire, which is divided into the same distinct 6 subscales: overall hand function, activities of daily living, pain (reversed), work performance, aesthetics, and patient satisfaction. The MHQ total score is obtained by averaging the scores for all 6 subscales. The total score ranges from 0 to 100 with a higher score indicating better hand performance. The ROM of fifth metacarpophalangeal joint was also recorded using a finger goniometer. The angulation was measured by the pronated oblique view of plain radiograph based on methods introduced and advocated in previous reports^12^,^13^. Briefly, the distal line was drawn from the mid-medullary point in the distal fragment to the most distal point of the metacarpal head, and the proximal line centrally through the shaft medullary canal, irrespective of where the line hit the basis of the metacarpal (Fig. 2).

Statistical analysis was performed using SPSS 22.0 software. A *P* value of <0.05 was considered to be statistically significant. Data were presented as mean ± standard deviation. Student’s t test and chi-square test were used to compare numeric and nonnumeric variables respectively.

## Results

Total 96 patients were included for analysis in our study. All of the treating surgeons were specialists of hand surgery. Anatomical reductions were achieved in all cases otherwise were excluded. Patients were divided based on the method of internal fixation. The average of follow-up period was 19 months (13 to 33 months). In our series, 61 of 96 cases were male. The average age of this series was 36.3 (20 to 59). The right hand was affected in 61, the left hand in 35 cases. Statistically, there was no significant difference of demographics between two groups. Generally, the clinical outcomes of internal fixation were satisfactory. The clinical outcomes one year after operations were shown in Table 1. The final angulation of plate group was 23.6 ± 9.7 and decreased to 11.8 ± 4.6 after the insertion of two crossed K-wires. The volar angulation immediately after surgery were similar between two groups (8.2 ± 2.9 versus 8.1 ± 3.5, *P* = 0.79). All patients with final angulation more than 30 degrees were from the plate group. As expected, the ROM of fifth metacarpophalangeal joint after K-wire fixation was superior compared to plate alone. Consistently, the MHQ score was significantly higher in plate with K-wire group.

There were no malunion, tendon rupture and neurological complications in all patients of this study. We also compared the rates of superficial infection and tenosynovitis between two groups (Table 2). There was no significant difference in the rates of superficial infection and tenosynovitis between two groups.

## Discussion

Different types of fixation for fifth metacarpal neck fractures have been described but none have gained consensus. Volar angulation was commonly observed after all current methods of fixation with immediate mobilization, which limited the efficacy of treatment. We hypothesized that insertion of two crossed K-wires after placement of locking plate could increase the strength of fixation, which prevents the secondary displacement and further improves the clinical outcomes.

In this study, we showed that locking plate in combination with two crossed K-wires could improve the clinical outcomes in fracture of fifth metacarpal neck, especially in range of motion. We assumed that the secondary angulation after surgery might be a vital cause of functional limitation in locking plate fixation. Although biomechanical study has shown that final angulation larger than 30 degrees could lead to functional limitation^2^, there is no clinical evidence to support this notion. Our study might partially support this theory since the major radiographic advance after K-wire fixation was the decreased angulation.

There were no neurological complications in all patients despite the extensive approach facing the dorsal cutaneous branch of the ulnar nerve. Nor did we encounter tendon rupture. In addition, there were no cases of malunion requiring revision, although this has been reported several times elsewhere^14^.

The major limitation of this study was that the study was its observational nature. The usages of K-wires were based on the surgeons’ preferences instead of randomization. Since all surgeries were conducted by specialists in hand surgery and the procedures of reduction and fixation with locking plate were well-standardized, the bias introduced by different surgeons might be mild. Supporting this notion, the average angulations of the two groups immediately after surgery were similar indicating the reduction quality was close. In addition, the two groups were well-matched. Thus, the major conclusion of this study was not affected.

## Conclusion

Locking plate in combination with two crossed K-wires is a more optimal method of fixation compared with locking plate alone.

## Additional Information

**How to cite this article**: Zhu, H. *et al*. Locking Plate Alone versus in Combination with Two Crossed Kirschner Wires for Fifth Metacarpal Neck Fracture. *Sci. Rep.* **7**, 46109; doi: 10.1038/srep46109 (2017).

**Publisher's note:** Springer Nature remains neutral with regard to jurisdictional claims in published maps and institutional affiliations.

## Figures and Tables

**Table 1:** Demographics Characteristics and Clinical outcomes at one year.

	Plate (n = 51)	Plate with K-wire (n = 45)	*P* Value
Male	32	29	0.75
Female	19	16	
Age	36.4 ± 9.8	36.2 ± 9.4	0.13
MHQ score	86.6 ± 7.1	96.7 ± 3.7	0.01
Grip strength	90.5 ± 4.7%	91.5 ± 5.3%	0.29
Angulation	23.6 ± 9.7	11.8 ± 4.6	0.01
ROM	72.2 ± 9.6	83.3 ± 7.3	0.01

ROM = active ROM of fifth metacarpophalangeal joint.

**Table 2:** Complications of each group.

	Plate (n = 51)	Plate with K-wire (n = 45)	*P* Value
Superficial infection	8	7	0.99
Tenosynovitis	2	2	0.90

Figures are numbers (percentage). N/A = Not applicable.
